# Supplementary material for: Conofolidine: A Natural Plant Alkaloid That Causes Apoptosis and Senescence in Cancer Cells
Source: Molecules. 2024 Jun 4;29(11):2654. doi: 10.3390/molecules29112654 (PMC11173856; doi:10.3390/molecules29112654)
Supplement: Supplementary file 1 [file molecules-29-02654-s001.zip › molecules-2831252-supplementary.pdf]

Conofolidine, a natural plant alkaloid causes apoptosis and senescence in cancer cells

Mohammed Zuhair Al-Hayali [1,*], Choy-Eng Nge [2], Kuan Hon Lim [3], Hilary M. Collins [4], Toh-Seok Kam [5] and Tracey D. Bradshaw [4,*]

1 Al-Kitab University, Kirkuk, 36015, Iraq
2 Department of Chemistry, Faculty of Science, University of Malaya, 50603 Kuala Lumpur, Malaysia; choy-eng86@gmail.com (C.E.N.); tskam@um.edu.my (T.S.K.)
3 School of Pharmacy, University of Nottingham Malaysia, Jalan Broga, 43500 Semenyih, Selangor Darul Ehsan, Malaysia; kuanhon.lim@nottingham.edu.my (K.H.L.)
4 School of Pharmacy, Biodiscovery Institute, University of Nottingham, University Park, Nottingham NG7 2RD, UK; hilary.collins@nottingham.ac.uk (H.C.)
* Correspondence: mohammed.zuhair@uoalkitab.edu.iq (M.A.H.); tracey.bradshaw@nottingham.ac.uk (T.D.B.)

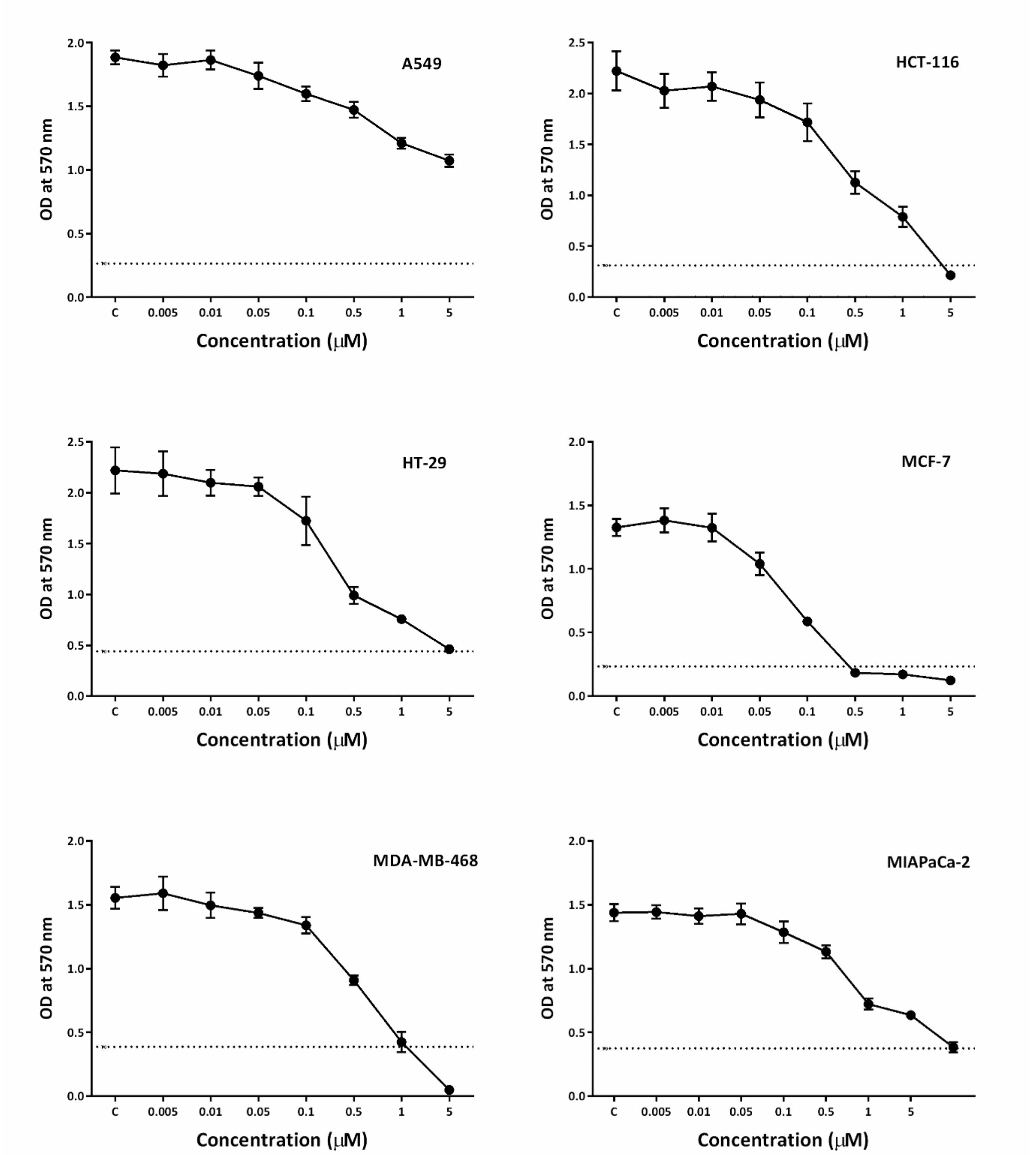

Figure S1. Growth inhibitory effects of conophylline from one independent trial in A549, HCT-116, HT-29, MCF-7, MDA-MB-468 and MIAPaCa-2 cells. Cells were seeded in 96-well plates at a density of 3×10^3 cells/well. After allowing to adhere (24 h), cells were treated with conophylline and incubated for 72 h. No. trials ≥3; n=4 per independent experiment.

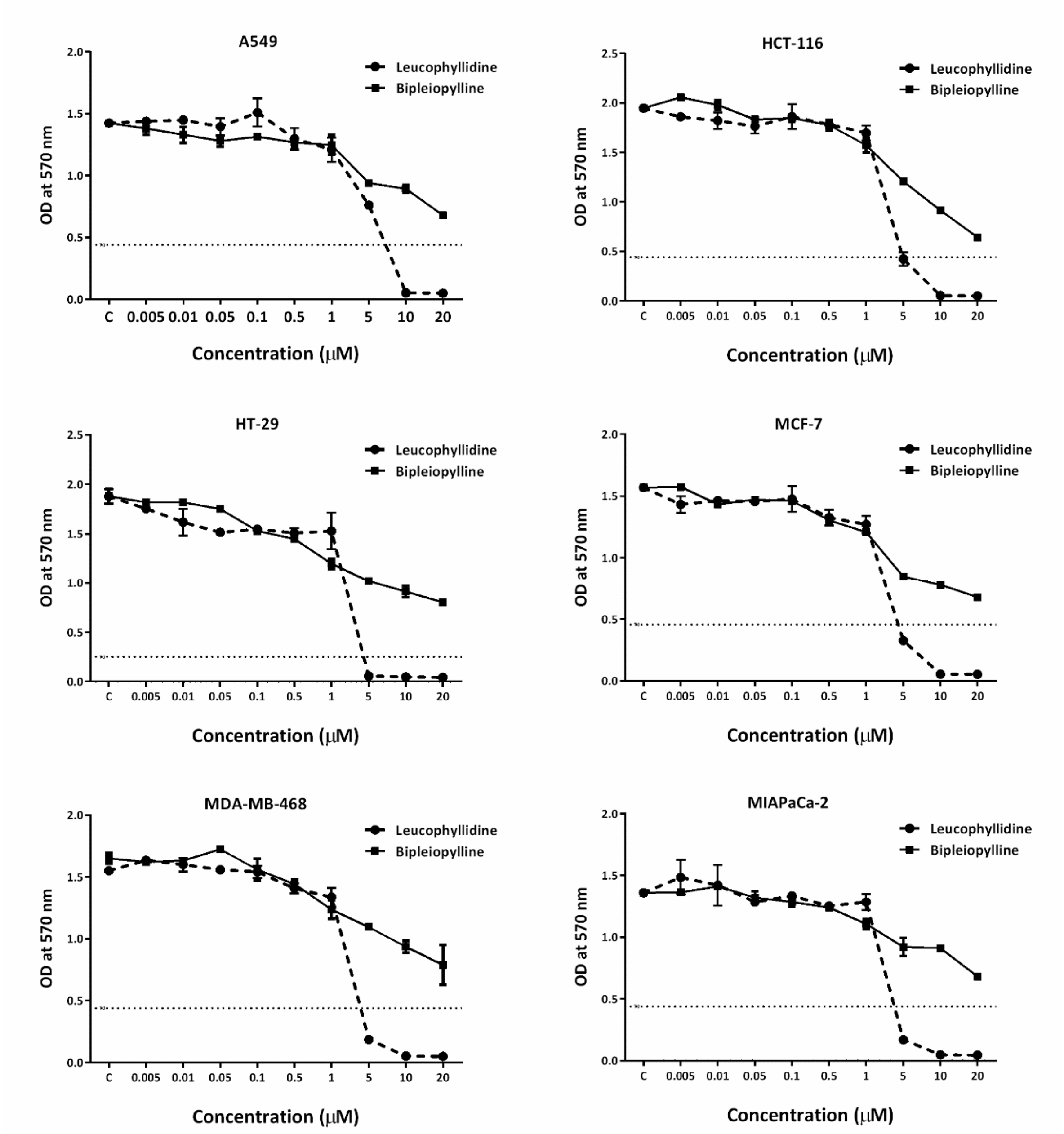

Figure S2. Growth inhibitory effects of leucophyllidine and bipleiophylline from independent trial in A549, HCT-116, HT-29, MCF-7, MDA-MB-468 and MIA PaCa-2 cells. Cells were seeded in 96-well plates at a density of 3×10^3 cells/well. After allowing to adhere (24 h), cells were treated with leucophyllidine and bipleiophylline and incubated for 72 h. No. trials ≥3; n=4 per independent experiment.

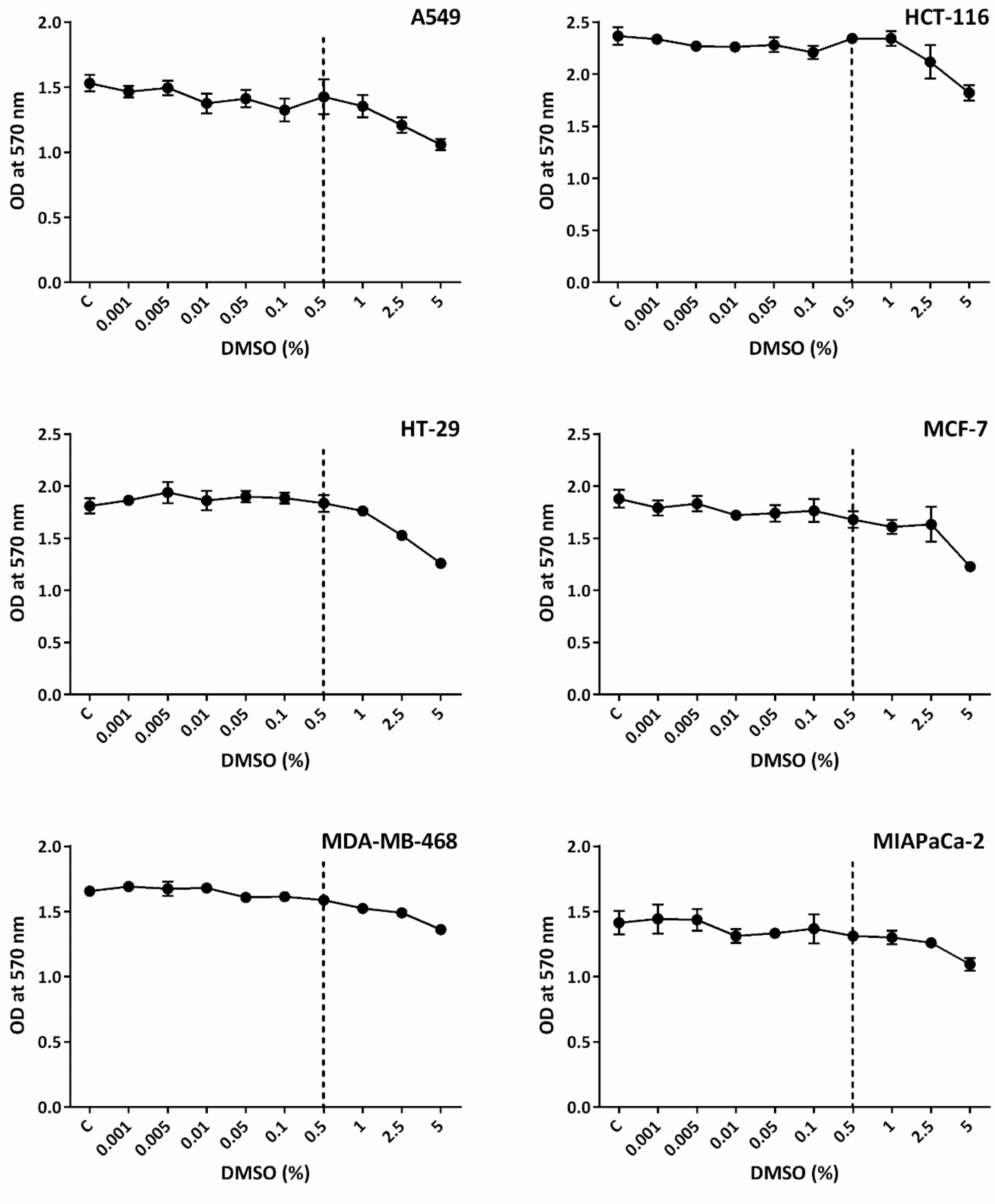

Figure S3. Line graphs show DMSO effects on the growth of A549, HCT-116, HT-29, MCF-7, MDA-MB-468 and MIAPaCa-2. Each representative graph shows one independent MTT trial. DMSO (%) is plotted against optical density. Cells were cultured for 24 h in 96-well plates (3×10^3 cells/180 µL medium/ well) then treated with DMSO alone for 72 h. Dashed line shows DMSO 0.5% treatment group (which corresponds to 0.5 µM conofolidine concentration) at which no significant growth inhibition was seen with DMSO alone in all cell lines. No. trials ≥3; n=4 per independent experiment.

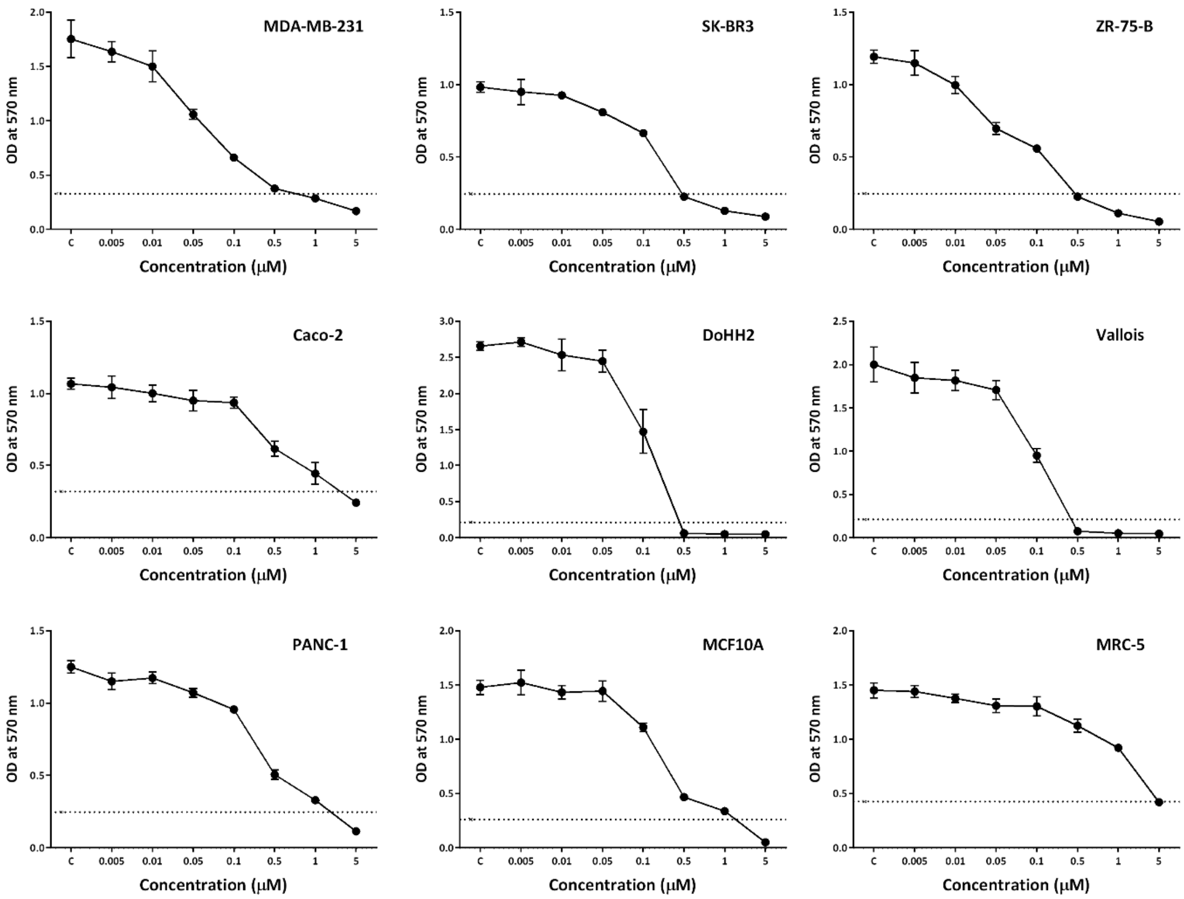

Figure S4. Growth inhibitory effects of conofolidine from one independent trial in MDA-MB-231, SK-BR3, ZR-75-B, Caco-2, Vallois, DoHH2, PANC1, MCF10A and MRC-5 cells. Cells were seeded in 96-well plates at a density of 3×10^3 cells/well. After allowing to adhere (24h), cells were treated with conofolidine and incubated for 72h. No. trials ≥3; n=4 per independent experiment.

Table S1. Antiproliferative effects of conofolidine on human cancer cells, benign fibrocystic epithelial breast cells and foetal lung fibroblasts.

**Human cell line**		**72h exposure MTT GI_{50} (µM)**
**Origin**	**Designation**	**Conofolidine**
Breast carcinoma	MDA-MB-231	0.051±0.01
	SK-BR3	0.168±0.03
	T-47D	0.117±0.04
	ZR-75-B	0.063±0.02
Colon carcinoma	Caco-2	0.417±0.02
Pancreatic carcinoma	PANC-1	0.312±0.04
Lymphoma (B-cell)	DoHH2	0.127±0.04
	Vallois	0.122±0.05
proliferative benign fibrocystic epithelial breast cells	MCF10A	0.225±0.03
Foetal lung fibroblast	MRC-5	0.992±0.05

GI_{50}s were estimated from MTT data after 72 h exposure to conofolidine (n= 4) and shown as mean ± SD of 3 independent trials

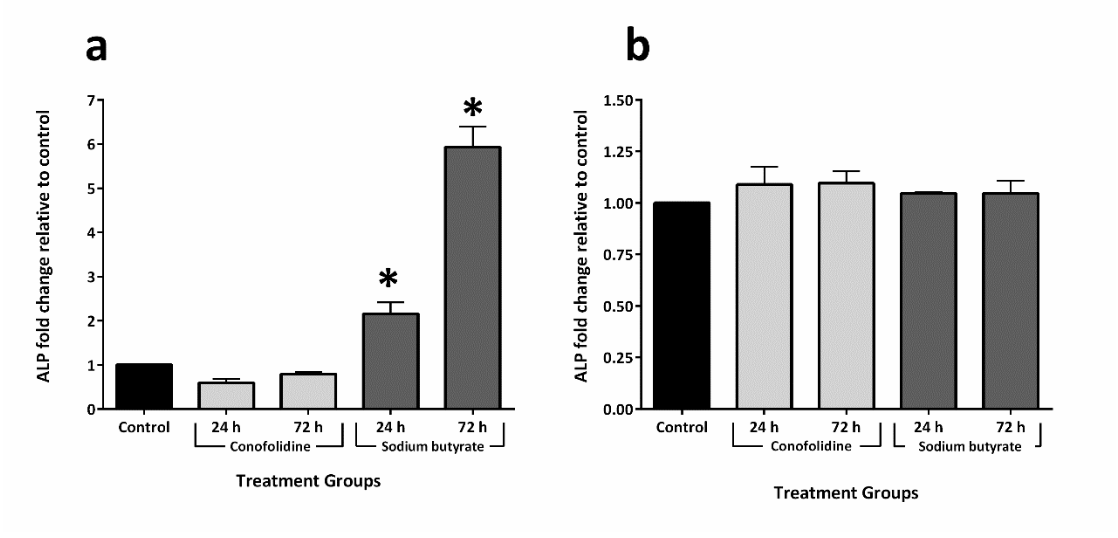

Figure S5. Assessment of alkaline phosphatase activity in HT-29 cells protein lysates and medium following 24 and 72 h treatments with conofolidine (2 x GI_{50}) and sodium butyrate (7.5 mM) compared to untreated control. (**a**) Mean bars ± SD of ALP activity in protein lysates; (**b)** Mean bars ± SD of ALP activity in HT-29 cells` media following conofolidine and sodium butyrate treatment compared to untreated control. Conofolidine treatment for 24 and 72 h did not increase ALP activity compared to untreated control in both protein lysates and medium. Sodium butyrate significantly caused time-dependent increase in ALP activity > 2-fold after 24 h and > 6-fold after 72 h exposures compared to untreated control in protein lysates; however, no significant increase was seen in medium. Asterisks indicate significant ($p \leq 0.05$) changes compared to control. Cells were seeded, allowed to attach overnight, then incubated with either vehicle alone, conofolidine or sodium butyrate. After 72 h, collection and measurement of absorbance were performed for both HT-29 cells protein lysates and medium at 405 nm. No. trials ≥3; n=2 per independent experiment.

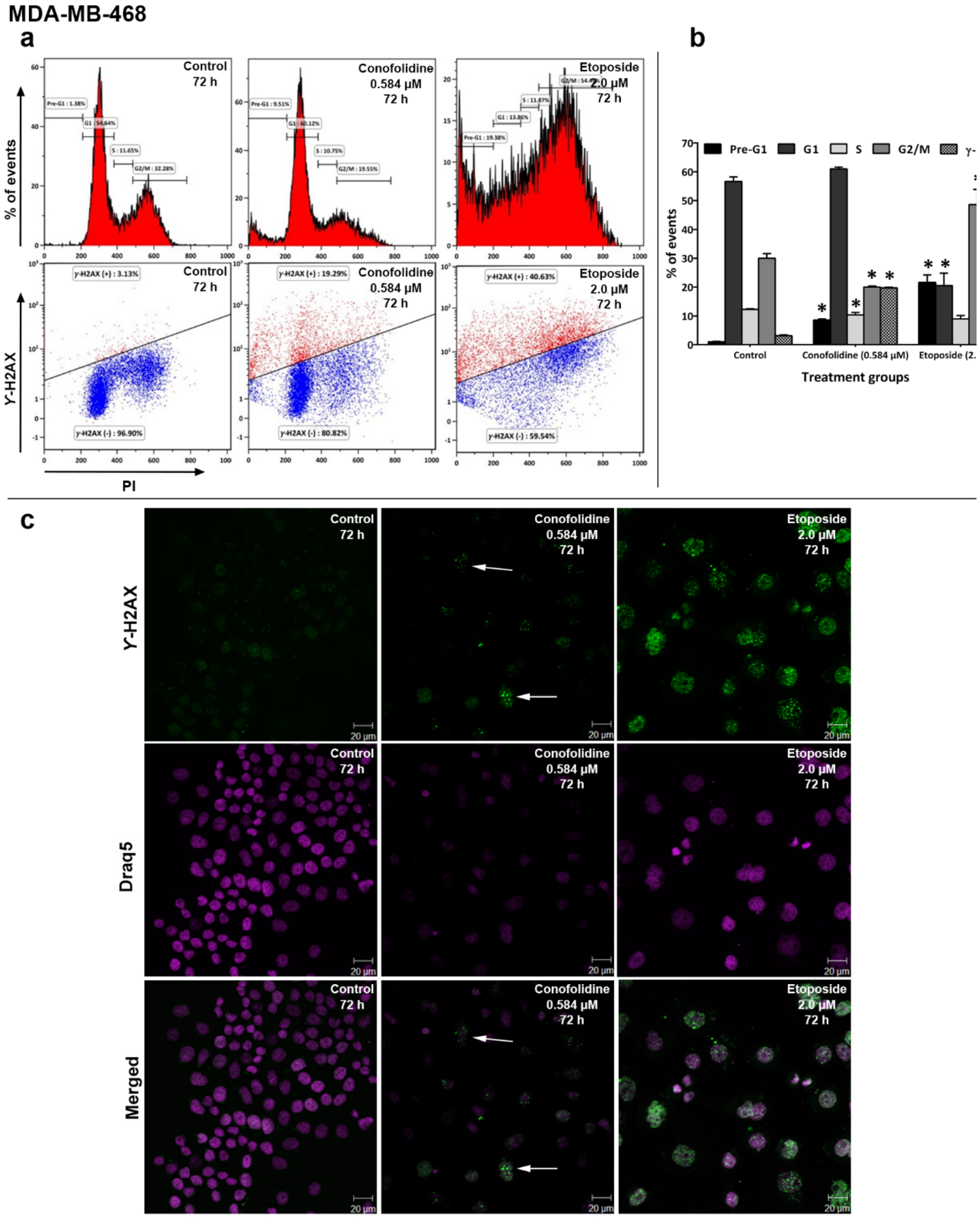

Figure S6. (a) Representative histograms and dot-plots from independent trial showing MDA-MB-468 cell cycle perturbation and γ-H2AX induction after 72h, 2xGI$_{50}$ conofolidine and etoposide treatments. The majority of DSBs was seen in G1 phase of cell cycle compared to controls and etoposide treated cells. **(b)** Mean±SD bars showing changes in percentage of MDA-MB-468 cell cycle phases and increased DNA-DSBs following 72h, 2xGI$_{50}$ conofolidine exposure compared to untreated control. Asterisks indicate significant ($p \leq 0.05$) changes. No. trials ≥3; n=2 per independent experiment. At least 15000 events were obtained/sample. **(c)** Confocal images from one trial of MDA-MB-468 treated with conofolidine showing γ-H2AX foci (green fluorescence) compared to control. Etoposide-treated cells also showed γ-H2AX foci formation. Arrows show examples of nuclei containing γ-H2AX foci saturation. Less DNA content was seen in each of conofolidine-treated cells compared to control. The experiment was repeated twice and at least three images were obtained from each treatment.

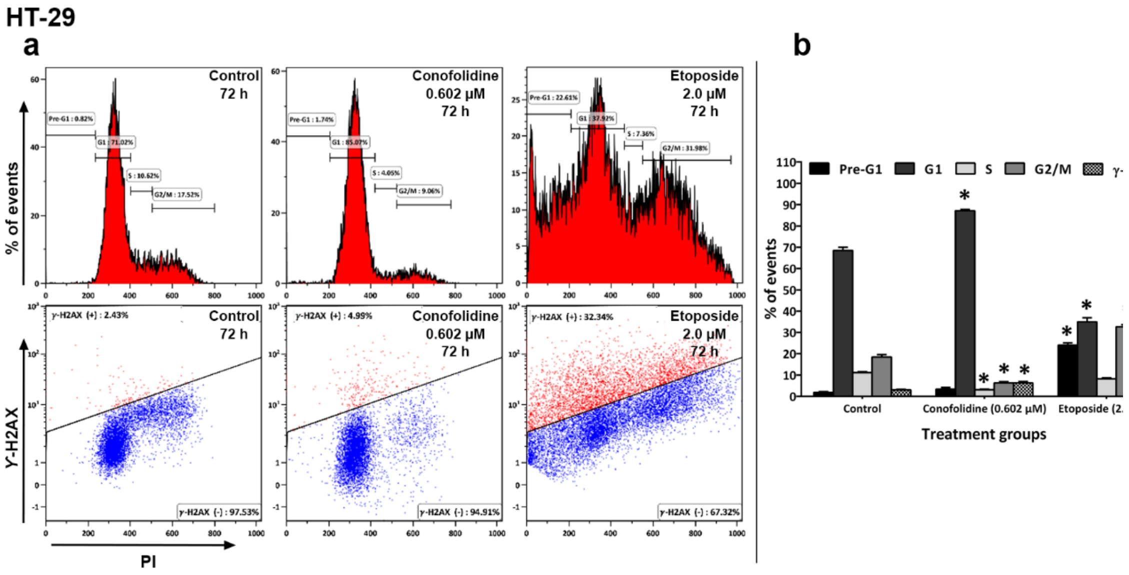

Figure S7. (a) Representative histograms and dot-plots from independent trial showing HT-29 cell cycle perturbation and γ-H2AX induction after 72h, 2xGI50 conofolidine and etoposide treatments. HT-29 cells showed the lowest percentage of DSBs compared to other cell lines. The majority of them were seen at G1 phase of cell cycle **(b)** Mean±SD bars showing changes in percentage of HT-29 cell cycle phases and increased DNA-DSBs following 72h, 2xGI50 conofolidine exposure compared to untreated control and etoposide. Asterisks indicate significant (p≤0.05) changes. No. trials ≥3; n=2 per independent experiment. At least 15000 events were obtained/sample.

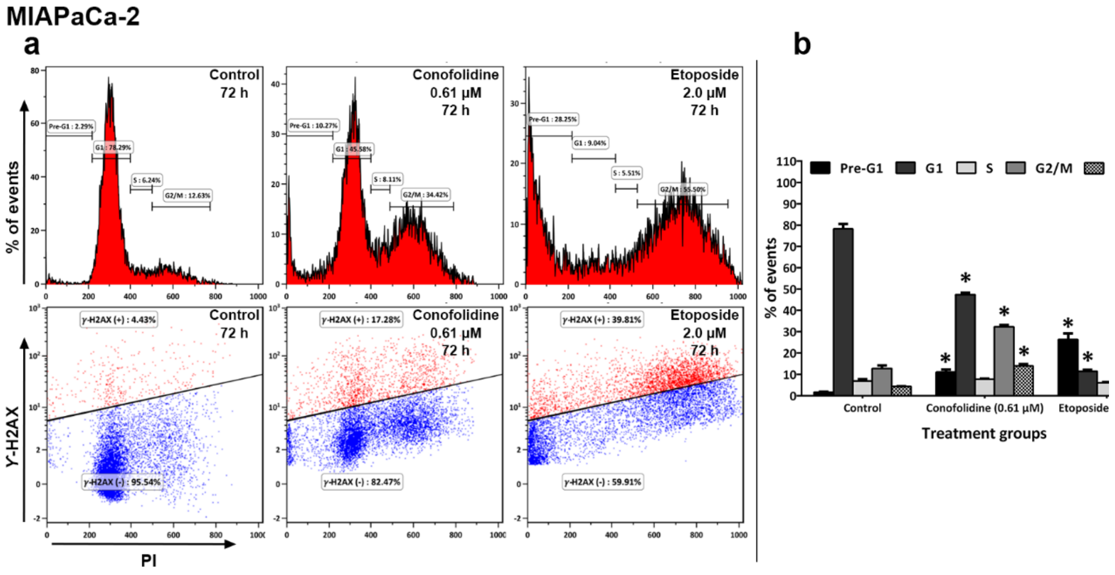

Figure S8. (a) Representative histograms and dot-plots from independent trial showing MIAPaCa-2 cell cycle perturbation and γ-H2AX induction after 72h, 2xGI50 conofolidine and etoposide treatments. MIAPaCa-2 cells showed DSBs in both G1 and G2/M phase of cell cycle. **(b)** Mean±SD bars showing changes in percentage of MIAPaCa-2 cell cycle phases and increased DNA-DSBs following 72h, 2xGI50 conofolidine exposure compared to untreated control and etoposide. Asterisks indicate significant (p≤0.05) changes. No. trials ≥3; n=2 per independent experiment. At least 15000 events were obtained/sample.
